# Identification of Chemical Toxicity Using Ontology Information of Chemicals

**DOI:** 10.1155/2015/246374

**Published:** 2015-10-05

**Authors:** Zhanpeng Jiang, Rui Xu, Changchun Dong

**Affiliations:** School of Software, Harbin University of Science and Technology, Harbin 150080, China

## Abstract

With the advance of the combinatorial chemistry, a large number of synthetic compounds have surged. However, we have limited knowledge about them. On the other hand, the speed of designing new drugs is very slow. One of the key causes is the unacceptable toxicities of chemicals. If one can correctly identify the toxicity of chemicals, the unsuitable chemicals can be discarded in early stage, thereby accelerating the study of new drugs and reducing the R&D costs. In this study, a new prediction method was built for identification of chemical toxicities, which was based on ontology information of chemicals. By comparing to a previous method, our method is quite effective. We hope that the proposed method may give new insights to study chemical toxicity and other attributes of chemicals.

## 1. Introduction

In drug discovery, detecting the toxicity of candidate drugs is a very important procedure. Some approved drugs such as phenacetin [1] and troglitazone [2], which have passed Phase III clinical trials, have to be withdrawn from the market, because their unexpected toxicities were detected. Pharmaceutical companies thus lost millions of dollars. In view of this, it is necessary to detect the toxicity of chemicals before they are selected as candidate drugs. However, evaluating the toxicity of a certain chemical requires comprehensive experimental testing, which costs millions of dollars and takes many years. On the other hand, with the advance of the combinatorial chemistry, a large number of synthetic compounds have surged, inducing that detecting chemical toxicities through traditional methods is an impossible task. Thus, quick, effective, and non-animal-involved prediction methods are urgently necessary.

In recent years, some prediction methods have been built for detecting chemical toxicities. Most of them can only deal with a single toxicity at the same time [3, 4], that is, predict a certain chemical to be toxic or nontoxic for a single toxicity. To detect all toxicities of a chemical, these methods have to be executed many times. Recently, Chen et al. built a multiclass prediction method using chemical-chemical interaction information [5], which can provide a candidate toxicity sequence ranging from the most likely toxicity to the least likely one. Their method was applied to detect the toxicities of chemicals listed in Accelrys Toxicity Database [6], in which six types of toxicity are reported: (1) acute toxicity; (2) mutagenicity; (3) tumorigenicity; (4) skin and eye irritation; (5) reproductive effects; (6) multiple dose effects. In this study, we employed the data in Chen et al.'s study [5] and adopted a new kind of information of chemicals to identify chemical toxicities. ChEBI ontology, integrated in a well-known database ChEBI (Chemical Entities of Biological Interest) [7], reports the ontology information of chemicals and is composed of the following subontologies: (1) molecular structure; (2) biological role; (3) application; (4) subatomic particle. Since gene ontology [8], the ontology information for proteins has been deemed to be a useful tool to investigate protein-related problems [9–12]. It is believed that ChEBI ontology is also a useful tool for studying chemicals and building effective prediction methods to identify chemical attributes. Here, we established a prediction method based on this information and compared to the method reported in [5]. The results indicate that this information is suitable to identify chemical toxicity. And we hope that the proposed method may stimulate extensive investigation based on this information, thereby promoting the study of chemicals and drug discovery.

## 2. Materials and Methods

### 2.1. Dataset

The toxicity information of chemicals was retrieved from a previous study [5], which was collected from the Accelrys Toxicity Database [6]. Six types of toxicity are reported in this database; there are (1) acute toxicity; (2) mutagenicity; (3) tumorigenicity; (4) skin and eye irritation; (5) reproductive effects; (6) multiple dose effects. Thus, the toxic chemicals in Accelrys Toxicity Database can be assigned to six classes. To investigate the problem of predicting chemical toxicity more throughout, we also employed the nontoxic chemicals, which were also retrieved from Chen et al.'s study [5]. These chemicals were collected from DrugBank (http://www.drugbank.ca/) [13] and Human Metabolome database (HMDB) (http://www.hmdb.ca/) [14]. Totally, 174,137 chemicals were collected and each of them was nontoxic or had at least one type of toxicity.

**Figure 1:**
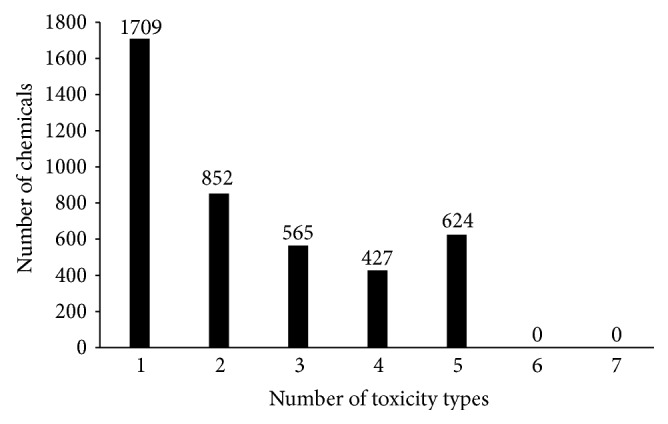
A histogram illustrating the number of chemicals having 1–7 types of toxicity.

To obtain a well-defined dataset, the chemicals with no ontology information were excluded, resulting in 4,177 chemicals. Thus, we obtained a dataset **S** consisting of 4,177 chemicals, in which 3,769 chemicals were toxic and 408 chemicals were nontoxic. As mentioned in the above paragraph, each toxic chemical has at least one type of toxicity. For convenience, let us tag the six types of toxicity using *t*_1_, *t*_2_,…, *t*_6_ and nontoxicity using *t*_7_. Accordingly, the dataset **S** can be separated into seven subsets formulated by

(1) $$\begin{array}{c} S=S_{1}\cup S_{2}\cup S_{3}\cup S_{4}\cup S_{5}\cup S_{6}\cup S_{7}, \end{array}$$

where **S**_*i*_ consisted of chemicals having toxicity *t*_*i*_. The number of chemicals in each subset (i.e., number of chemicals having each type of toxicity) is listed in Table 1, column 3, from which we can see that the acute toxicity was a greatest type of toxicity containing most chemicals, followed by mutagenicity, multiple dose effects, and so forth, while the number of nontoxic chemicals was least. Since some chemicals may have more than one type of toxicity, that is, they may occur in more than one set of **S**_1_, **S**_2_,…, **S**_6_, the sum of numbers in seven subsets was larger than the total number of chemicals in **S**. Thus, it is a multilabel classification problem.  Figure 1 gives the number of chemicals having 1–7 types of toxicity. Like many previous studies dealing with multilabel classification problem [5, 15, 16], the proposed method would give a series of candidate toxicities for each query chemical with the sequence from most likely toxicity to the least likely one.

### 2.2. Construction of a Graph by Ontology Information of Compound

The ontology information of compound was retrieved from ChEBI (http://www.ebi.ac.uk/chebi/init.do) [7]. We downloaded a file named as “chebi.obo” (accessed November 2014) from its ftp website: ftp://ftp.ebi.ac.uk/pub/databases/chebi/ontology/, which contains larger number of ontology terms and their descriptions. Since the ontology terms can be conceived as graph-theoretical structures, a graph can be constructed according to the information of all ontology terms, in which nodes represent ontology terms and edges denote the relationship between two terms. By using the entries “is a” and “relationship” in the obtained file to indicate the relationship between two terms, we constructed a large graph *G* with 45,206 nodes and 113,549 edges.

### 2.3. Prediction Method

As mentioned in Section 2.2, a graph was constructed according to the ontology information of compounds. It can be observed that the corresponding ontology terms of two adjacent nodes in *G* have some special relationship. And it can be further inferred that if two nodes are with small distance in *G*, the corresponding ontology terms have close linkage. In view of this, using the distance in *G* to quantitatively measure the relationship between two ontology terms is reasonable. For two terms *a*_1_ and *a*_2_, let us denote the distance of the corresponding nodes in *G* by *d*(*a*_1_, *a*_2_).

For two chemicals *c*_1_ and *c*_2_, let *a*_11_, *a*_12_,…, *a*_1*k*_ be the ontology terms of *c*_1_ and let *a*_21_, *a*_22_,…, *a*_2*l*_ be the ontology terms of *c*_2_. It is obvious that if *d*(*a*_1*i*_, *a*_2*j*_) (1 ≤ *i* ≤ *k*, 1 ≤ *j* ≤ *l*) is small, *c*_1_ and *c*_2_ are highly related and have high probability to share same structures, functions, and so on. Thus, we gave the following formulation to measure the common features of chemicals *c*_1_ and *c*_2_:

(2) $$\begin{array}{c} S\left(c_{1},c_{2}\right)=\min\left\{d\left(a_{1i},a_{2j}\right):1\leq i\leq k,\;\;1\leq j\leq l\right\}, \end{array}$$

where *d*(*a*_1*i*_, *a*_2*j*_) denote the distance of terms *a*_1*i*_ and *a*_2*j*_ in the graph constructed in Section 2.2, which can be obtained by Dijkstra's algorithm [17]. The smaller the *S*(*c*_1_, *c*_2_) is, the closer the relationship *c*_1_ and *c*_2_ have.

The proposed prediction method highly relied on the result of (2). To introduce the method clearly, it is necessary to employ some notations. Let **S**′ be a training set consisting of *n* chemicals, say *c*_1_, *c*_2_,…, *c*_*n*_; that is, **S**′ = {*c*_1_, *c*_2_,…, *c*_*n*_}. The toxicity information of each *c*_*i*_  (1 ≤ *i* ≤ *n*) can be represented by

(3) $$\begin{array}{c} T\left(c_{i}\right)={\left[b_{i1},b_{i2},\ldots ,b_{i7}\right]}^{T}\;\left(1\leq i\leq n\right), \end{array}$$

where *b*_*ij*_  (1 ≤ *j* ≤ 7) was defined by

(4) $$\begin{array}{c} b_{ij}=\left\{\begin{array}{cc} 1 & \text{if}\;c_{i}\;\text{has}\;\text{toxicity}\;t_{j}\\ 0 & \text{otherwise}. \end{array}\right. \end{array}$$

For a query chemical *c*, its score of having toxicity *t*_*j*_ was calculated as follows.

(1) For each chemical *c*_*i*_ in the training set **S**′, calculate *S*(*c*, *c*_*i*_) according to (2). Then, find all nearest neighbors, say *c*_1_, *c*_2_,…, *c*_*k*_, without generalization, such that *S*(*c*, *c*_*i*_) = min⁡{*S*(*c*, *c*′) : *c*′ ∈ **S**′}  (1 ≤ *i* ≤ *k*).
(2) For each *t*_*j*_, the score of *c* having toxicity *t*_*j*_ was calculated by
  (5) $$\begin{array}{c} P\left(c⊳t_{j}\right)=\overset{k}{\underset{i=1}{\sum }}b_{ij}. \end{array}$$

It is easy to observe that the score of *c* having toxicity *t*_*j*_ is the number of chemicals among *c*_1_, *c*_2_,…, *c*_*k*_ which have toxicity *t*_*j*_. Since *c*_1_, *c*_2_,…, *c*_*k*_ are highly related to *c*, larger *P*(*c*⊳*t*_*j*_) indicates that many closely related training chemicals of *c* have toxicity *t*_*j*_, inducing that the probability of *c* having toxicity *t*_*j*_ is high. In particular, *P*(*c*⊳*t*_*j*_) = 0 suggests that the score of *c* having toxicity *t*_*j*_ is zero, inducing that the possibility of *c* having this toxicity is zero.

As mentioned in Section 2.1, the investigated problem is a multilabel classification problem. Only giving the most likely candidate toxicity is not enough. Fortunately, we can output a series of candidate toxicities according to the scores of the query chemical having 7 types of toxicity. The toxicity which receives the highest score is the most likely toxicity, while the toxicity receiving the second highest score is the second likely toxicity and so forth. For example, if the rank of seven scores for a certain query chemical *c* is

(6) Pc⊳t1≥P(c⊳t4)≥P(c⊳t2)>P(c⊳t3)=P(c⊳t5)=P(c⊳t6)=P(c⊳t7)=0,

it suggests *t*_1_ (i.e., acute toxicity) is the most likely toxicity for *c*, followed by *t*_4_ (i.e., skin and eye irritation) and *t*_2_ (i.e., mutagenicity), while the other types of toxicity are not predicted to be candidate toxicities for *c*. Furthermore, *t*_1_ is called the first prediction, *t*_4_ the second prediction, and so forth.

### 2.4. Accuracy Measurements

For a query chemical, the proposed method can provide a series of candidate toxicities. In view of this, we should calculate the accuracy for each order prediction. The *k*th prediction accuracy can be computed by [5, 15]

(7) $$\begin{array}{c} {\text{ACC}}_{k}=\frac{CP_{k}}{N}\;k=1,2,\ldots ,7, \end{array}$$

where *CP*_*k*_ is the number of chemicals whose *k*th prediction is correct and *N* is the total number of chemicals that are predicted by the method. Since it is difficult to know the number of toxicities for a query chemical, the first prediction accuracy is the most important measure to evaluate the performance of the method. In addition, an effective prediction method for a multilabel classification problem should rank the candidate toxicities well; that is, prediction accuracies should follow a decreasing trend with the increasing of the prediction order.

Besides, to evaluate the performance of prediction method on the whole, another measurement was also adopted [5, 15]. It measures the proportion of the true toxicities covered by the first *m* predictions of chemicals, which can be calculated by

(8) $$\begin{array}{c} W_{m}=\frac{\sum_{i=1}^{N}\Psi_{i}^{m}}{N_{i}}, \end{array}$$

where Ψ_*i*_^*m*^ is the number of true toxicities of the *i*th chemical which are listed among its first *m* predictions and *N*_*i*_ is the total number of true toxicities of the *i*th chemical. Generally, *m* is always taken as the smallest integer bigger than or equal to the average number of toxicities of chemicals processed by the method; that is, *m* = ⌈∑_*i*=1_^*N*^ *N*_*i*_/*N*⌉. It is obvious that larger *W*_*m*_ indicates the true toxicities are arranged in the front of candidate toxicities.

## 3. Results and Discussion

### 3.1. Performance of the Method

For the 4,177 chemicals in **S**, the prediction method was executed to identify their toxicities evaluated by jackknife test [15]. The seven prediction accuracies thus obtained by (7) are listed in Table 2, column 2. It can be observed that the first prediction accuracy was 75.17%, the second one was 43.52%, and the third one was 28.47%. Furthermore, seven prediction accuracies always followed a decreasing trend with the increasing of the prediction order, indicating the proposed method arranged the candidate toxicities of all tested chemicals quite well. In addition, the average number of toxicities of chemicals in **S** was about 2.38. Thus, the first three predictions of all chemicals in **S** were collected, obtaining the accuracy of 61.87% by (8), which means the proportion of the true toxicities of chemicals in **S** covered by their first three predictions. All of these indicate that the proposed method is quite effective for identification of chemical toxicities.

### 3.2. Understanding the Method by Listing an Example

To better understand our method, this section listed an example. CID104975 is a chemical with toxicity *t*_2_ (mutagenicity) and *t*_3_ (tumorigenicity). Its ontology term is CHEBI:25957. According to the method, we computed the distance between CHEBI:25957 and ontology terms of other chemicals in **S**, thereby calculating the relationship between CID104975 and other chemicals by (2). Four chemicals, listed in Table 3, were found to be closely related to CID104975; they are CID995, CID2236, CID6763, and CID13257. Their toxicities and ontology terms are listed in Table 3, column 2 and column 3, respectively. By the method, the toxicity *t*_1_ received 3 votes, *t*_2_ 4 votes, *t*_3_ 3 votes, *t*_6_ 2 votes, and other toxicities no votes. Accordingly, we obtained that the candidate toxicities for CID104975 were *t*_2_, *t*_1_, *t*_3_, and *t*_6_. It is obvious that the first and third predictions were correct, while the second prediction was incorrect.

### 3.3. Comparison of Other Methods

In this section, we employed another kind of chemical information, which has been applied for identification of chemical toxicities in Chen et al.'s study [5]. Their method used chemical-chemical interaction information, which has been deemed to be useful information for study of chemical-related problems [5, 15, 18, 19], to build the prediction method, and gave good performance.

To compare our method and Chen et al.'s method in a fair circumstance, a chemical set, consisting of 3,955 chemicals, was extracted from **S**, called **S**_**c**_, such that each chemical in **S**_**c**_ has both ontology information and interaction information; that is, each chemical can be predicted by these two methods. The number of chemicals in **S**_**c**_ on each type of toxicity is listed in Table 1, column 4, from which we can see that the distribution of 3,955 chemicals on seven types of toxicity is similar to chemicals in **S**. Also some chemicals have two or more toxicities. Our method and Chen et al.'s method were all executed on **S**_**c**_ with their performance being evaluated by jackknife test. Listed in Table 2, columns 3 and 4, are seven prediction accuracies. It can be seen that the first prediction accuracy of our method was 75.40%, which is little higher than 75.14% of Chen et al.'s method. However, with the increasing of prediction order, the prediction accuracies of Chen et al.'s method were higher than those obtained by our method. It is reasonable because the ontology information of chemicals is not very complete at present, which induces that many relations of ontology terms have not been detected. Furthermore, we also calculated the measurement defined in (8). Since the average number of toxicities of chemical in **S**_**c**_ was about 2.44, the first three predictions of chemicals in **S**_**c**_, which were obtained by two methods, were collected, thereby obtaining the accuracy of 61.70% for our method and 65.31% for Chen et al.'s method. It is also caused by the aforementioned reason. Although, if one considers more than one toxicity for a certain chemical, our method is not better than Chen et al.'s method, the first prediction accuracy of our method is higher than that of Chen et al.'s method, which is the most important one because one always pays more attention to the most likely toxicity for a chemical. In view of this, we believe that our method has superiority for identification of chemical toxicities.

## 4. Conclusions

This study gave a new prediction method to identify chemical toxicities. By utilizing the ontology information of chemicals reported in ChEBI, one can predict the toxicities of a certain chemical with quite high quality. It is hopeful that this method may promote the study of chemicals.

## Figures and Tables

**Table 1:** Distribution of chemicals in **S** and **S**_**c**_.

Tag of toxicity	Type of toxicity	Number of chemicals in **S** ^a^	Number of chemicals in **S**_**c**_^b^
*t*_1_	Acute toxicity	3144	2993
*t*_2_	Mutagenicity	1850	1814
*t*_3_	Tumorigenicity	881	871
*t*_4_	Skin and eye irritation	954	935
*t*_5_	Reproductive effects	1099	1080
*t*_6_	Multiple dose effects	1600	1570
*t*_7_	Nontoxic	408	374

Total	—	9936	9637

^a^ **S** is a chemical set consisting of 4,177 chemicals, which was used to examine our method.

^b^ **S**_**c**_ is another chemical set consisting of 3,955 chemicals, which was used to compare our method with a previous method.

**Table 2:** Performance of the methods on **S** and **S**_**c**_.

Prediction order	Our method on **S** ^a^	Our method on **S**_**c**_^b^	Chen et al.'s method on **S**_**c**_^b^
1st	75.17%	75.40%	75.14%
2nd	43.52%	45.18%	49.87%
3rd	28.47%	29.76%	34.11%
4th	23.34%	24.15%	29.94%
5th	16.78%	17.98%	27.00%
6th	9.74%	10.24%	19.97%
7th	3.16%	3.16%	5.54%

^a^ **S** is a chemical set consisting of 4,177 chemicals, which was used to examine our method.

^b^ **S**_**c**_ is another chemical set consisting of 3,955 chemicals, which was used to compare our method with a previous method.

**Table 3:** Chemicals with closest relationship of CID104975.

Compound ID	Tag of toxicity	Ontology information	Shortest path to CHEBI25957
CID995	*t*_1_, *t*_2_, *t*_3_, and *t*_6_	CHEBI:28851	CHEBI:25957, CHEBI:25959, CHEBI:25961, and CHEBI:28851
CID2236	*t*_1_, *t*_2_, *t*_3_, and *t*_6_	CHEBI:2825	CHEBI:25957, CHEBI:25959, CHEBI:25961, and CHEBI:2825
CID6763	*t*_1_, *t*_2_, and *t*_3_	CHEBI:37454	CHEBI:25957, CHEBI:25959, CHEBI:25961, and CHEBI:37454
CID13257	*t*_2_	CHEBI:35860	CHEBI:25957, CHEBI:25959, CHEBI:25961, and CHEBI:35860
